# Topic Modeling for Interpretable Text Classification From EHRs

**DOI:** 10.3389/fdata.2022.846930

**Published:** 2022-05-04

**Authors:** Emil Rijcken, Uzay Kaymak, Floortje Scheepers, Pablo Mosteiro, Kalliopi Zervanou, Marco Spruit

**Affiliations:** ^1^Jheronimus Academy of Data Science, Eindhoven University of Technology, Eindhoven, Netherlands; ^2^Department of Information and Computing Sciences, Utrecht University, Utrecht, Netherlands; ^3^University Medical Center Utrecht, Utrecht, Netherlands; ^4^Public Health and Primary Care (PHEG), Leiden University Medical Center, Leiden University, Leiden, Netherlands; ^5^Leiden Institute of Advanced Computer Science (LIACS), Faculty of Science, Leiden University, Leiden, Netherlands

**Keywords:** text classification, topic modeling, explainability, interpretability, electronic health records, psychiatry, natural language processing, information extraction

## Abstract

The clinical notes in electronic health records have many possibilities for predictive tasks in text classification. The interpretability of these classification models for the clinical domain is critical for decision making. Using topic models for text classification of electronic health records for a predictive task allows for the use of topics as features, thus making the text classification more interpretable. However, selecting the most effective topic model is not trivial. In this work, we propose considerations for selecting a suitable topic model based on the predictive performance and interpretability measure for text classification. We compare 17 different topic models in terms of both interpretability and predictive performance in an inpatient violence prediction task using clinical notes. We find no correlation between interpretability and predictive performance. In addition, our results show that although no model outperforms the other models on both variables, our proposed fuzzy topic modeling algorithm (FLSA-W) performs best in most settings for interpretability, whereas two state-of-the-art methods (ProdLDA and LSI) achieve the best predictive performance.

## 1. Introduction

Inpatient violence at psychiatry departments is a common and severe problem (van Leeuwen and Harte, 2017). Typical adverse reactions that victims (professionals) face include emotional reactions, symptoms of post-traumatic stress disorder, and a negative impact on work functioning. Therefore, it is vital to assess the risk of a patient showing violent behavior and take preventive measures. The psychiatry department of the Utrecht Medical Center Utrecht uses questionnaires to predict the likelihood of patients becoming violent. However, filling out these forms is time-consuming and partly subjective. Instead, automated machine-learning approaches based on existing patient information could overcome the time burden and help make more objective predictions. Various automated text classification approaches utilizing clinical notes in the electronic health record allow for more accurate predictions than the questionnaires (Menger et al., 2018a; Mosteiro et al., 2021). In addition to accurate predictions, clinical providers and other decision-makers in healthcare consider the interpretability of model predictions as a priority for implementation and utilization. As machine learning applications are increasingly being integrated into various parts of the continuum of patient care, the need for prediction explanation is imperative (Ahmad et al., 2018). Yet, an intuitive understanding of the automated text classification approaches' inner workings is currently missing, as the clinical notes are represented numerically by large dense matrices with unknown semantic meaning.

A more intuitive and potentially interpretable approach is text classification through topic modeling, where clinical notes can be represented as a collection of topics. To do so, a topic model is trained on all the written notes to find *k* topics. Each topic consists of the *n* most likely words associated with that topic and weights for each word. After training the topic model, all the documents associated with one patient can be represented by a *k*-length vector in which each cell indicates the extent to which that topic appears in the text. The assumption is that if the generated topics are well interpretable, the model's decision making is more explainable. Several authors have focused on text classification through topic modeling in health care (Rumshisky et al., 2016; Wang et al., 2019). They use Latent Dirichlet Allocation (LDA) (Blei et al., 2003). Yet, many other topic modeling algorithms exist and selecting a model is not straightforward. A topic modeling algorithm for text classification should be selected based on predictive performance and interpretability. If a model performs well on predictions but is not interpretable, then there is no added value for our analysis in using topic models for this task. Similarly, if the predictive performance is low, but the interpretability is high, topic models should not be used for classification. We note that, to the best of our knowledge, no previous work focuses on both the predictive performance and interpretability of topic models for text classification.

In this article, we train seventeen different topic models and use their topic embeddings as input for text classification (violence prediction). Then, we analyze how each model's interpretability compares to its predictive value. From this analysis, we make the following contributions:

1. We are the first to analyze both the predictive performance and topic modeling interpretability.
2. We compare 17 topic modeling algorithms based on both criteria.
3. We present considerations that can be used for the selection of a topic model for text classification.

The outline of the article is as follows. In Section 2, we describe how topic models work, how they can be used for text classification, how different algorithms relate to one another and which measures are used for evaluation. In Section 3, we describe our comparison methodology and the data set that we used. In Section 4, we provide tables and show graphs to illustrate how different topic modeling algorithms compare to each other. In Section 5, we discuss our findings, its implications and we conclude the work in Section 6.

## 2. Topic Modeling Algorithms

We compare different topic modeling algorithms based on their interpretability and predictive performance for text classification. In this section, we describe the task of text classification, followed by a description of topic models. Then, we discuss the best-known topic modeling algorithms and discuss how these have been used for text classification.

### 2.1. Text Classification

Classification models are a set of techniques that map input data (in the feature space) to a fixed set of output labels (Flach, 2012). Text classification is the task of assigning such a label to a text. Typically, a ML text classification pipeline contains two steps:

1. representation step,
2. classification step.

In the first step, a text file is transformed from a string into a numeric representation, called an embedding. The classification algorithm in the next step then calculates the most likely label based on the embedding. The choice of the technique depends on various aspects such as the number of features, the size of the data set and whether a technique should be interpretable. Typically, classification models are considered to be interpretable if they can indicate the weights that have been assigned to each input feature. Amongst classification models, the subset of commonly used interpretable models include linear regression, logistic regression, decision trees, fuzzy systems, and association rules (Guillaume, 2001; Alonso et al., 2015).

#### 2.1.1. Representation Techniques

Early approaches for representing texts numerically used the bag-of-words approach (BOW) to represent each word as a one-hot-encoding (Jurafsky and Martin, 2009). BOW suffers from two significant limitations: (i) it is hard to scale, (ii) it only considers the presence of a word in a text and not the word's location. Therefore, it does not capture syntactic information.

Neural embeddings such as Word2Vec (Mikolov et al., 2013) do not suffer from BOW's limitations and have been used widely ever since being introduced in 2013. Through neural embeddings, words are represented as dense vectors in a high-dimensional space such that semantically similar words are located close to each other. Since WordsVec's introduction, several neural embedding approaches have been used for text classification, such as BERT (Devlin et al., 2018), Doc2Vec (Le and Mikolov, 2014), Glove (Pennington et al., 2014), and ELMO (Peters et al., 2018). These neural models have improved the performance of text classification significantly. However, relatively little is known about the information captured by these embeddings' features. Therefore, there is still little understanding of the classification decisions in the subsequent step. Alternatively, the topics trained by topic models could serve as features for text classification. These topics are better interpretable than the features in neural representations and could help understand text classification decisions better.

### 2.2. Topic Models

Topic models are a group of unsupervised natural language processing algorithms that calculate two quantities:

1. *P*(*W*_*i*_|*T*_*k*_)- the probability of word *i* given topic *k*,
2. *P*(*T*_*k*_|*D*_*j*_)- the probability of topic *k* given document *j*,

with:

*i* word index *i*∈{1, 2, 3, ..., *M*},

*j* document index *j*∈{1, 2, 3, ..., *N*},

*k* topic index *k*∈{1, 2, 3, ..., *C*},

*M* the number of unique words in the data set,

*N* the number of documents in the data set,

*C* the number of topics.

The top-*n* words with the highest probability per topic are typically taken to represent a topic. Topic models aim to find topics in which these top-*n* words in each topic are coherent with each other so that the topic is interpretable and a common theme can be derived. Using topic embeddings for text classification, each input document is transformed into a vector of size *C*. Each cell indicates the extent to which the document belongs to a topic. After predictions are made for each input text, interpretable classification algorithms can reveal which topics were most important for performing classifications.

### 2.3. Topic Modeling Algorithms

We compare a set of state-of-the-art topic modeling algorithms as defined in Terragni et al. (2021) supplemented with topic modeling algorithms we have developed in an earlier study (Rijcken et al., 2021). The different methods can be divided into two categories; methods based on dimensionality reduction and methods based on the Dirichlet distribution.

#### 2.3.1. Dimensionality Reduction Methods

The algorithms based on dimensionality reduction all start with a document-term matrix **A**. This could be a simple bag-of-words representation, but typically a weighting mechanism such as tf-idf is applied. The algorithms based on dimensionality reduction are the following.

##### 2.3.1.1. NMF

One of the oldest methods is non-negative matrix factorization (NMF) (Févotte and Idier, 2011). Using matrix **A**, NMF returns two matrices **W** & **H**. Since the vectors of the decomposed representations are non-negative, their coefficients are non-negative as well. **W** contains the found topics (topics × words) and **H** contains the coefficients (documents × topics). Then, NMF modifies **W** and **H**'s initial values so that its product approaches **A**.

##### 2.3.1.2. LSI

Other foundational work on topic modeling is latent semantic indexing (LSI)^1^ which uses singular value decomposition for dimensionality reduction on matrix **A** (Landauer et al., 1998). SVD's output is a decomposition of **A**, such that **A** = **UΣV**^*T*^. In this case, **U** emerges as the document-topic matrix ***P*(*T*_*k*_|*D*_*j*_)**, **V** becomes the term-topic matrix ***P*(*W*_*i*_|*T*_*k*_)** and **Σ** contains singular values in its diagonal.

##### 2.3.1.3. FLSA

Similar to LSA, fuzzy latent semantic analysis (FLSA) starts with matrix **A** and uses singular value decomposition for dimensionality reduction (Karami et al., 2018). FLSA hypothesizes that singular value decomposition projects words into a lower dimensional space in a meaningful way, such that words that are semantically related are located nearby each other. FLSA takes the **U** matrix from singular value decomposition (number of singular values × number of documents), then performs fuzzy c-means clustering (Bezdek, 2013) to find different topics and lastly uses Bayes' theorem and linear algebra to find the two output matrices.

##### 2.3.1.4. FLSA-W

Since FLSA works with the **U** matrix, which gives singular values for each document, the clustering is based on documents. Yet, topics are distributions over words and therefore clustering words seems to make more sense. Therefore, FLSA-W clusters on the **V** matrix instead of **U**, hence by clustering on words directly.

##### 2.3.1.5. FLSA-V

While FLSA and FLSA-W implicitly assume that the projection to a lower dimensional space occurs in a meaningful way, there is no explicit step guarantying it. FLSA-V uses a projection method similar to multi-dimensional scaling (Borg and Groenen, 2005) for embedding the words into a lower dimensional manifold such that similar words (based on co-occurrence) are placed close together on the manifold (Van Eck and Waltman, 2010). Then, the algorithm performs similar steps as FLSA-W to find the topics. We note that the projection step is very memory intensive and the implementation we need (VOSviewer software, Van Eck and Waltman, 2010) ran into memory issues with large corpuses and require heavy pruning to perform its mapping.

#### 2.3.2. Dirichlet-Based Models

##### 2.3.2.1. LDA

Underlying the class of dimensionality reduction methods that includes the prior models is the “BOW assumption,” which states that the order of words in a document can be neglected. The irrelevance of order also holds for documents, as it does not matter in what order documents occur in a corpus for a topic model to be trained. De Finetti's representation theorem (De Finetti, 2017) establishes that any collection of exchangeable random variables has a representation as a mixture distribution. Thus, to consider exchangeable representations for words and documents, mixture models that capture the exchangeability of both should be used. This line of thought paves the way to Latent Dirichlet Allocation (LDA) (Blei et al., 2003), which is the best-known topic modeling algorithm on which multiple other topic models are based. LDA posits that each document can be seen as a probability distribution over topics and that each topic can be seen as a probability distribution over words. From a Dirichlet distribution, which is a multivariate generalization of the beta distribution, a random sample is drawn to represent the topic distribution. Then, a random sample is selected from another Dirichlet distribution to represent the word distribution.

##### 2.3.2.2. ProdLDA and NeuralLDA

Although the posterior distribution is intractable for exact inference, many approximate inference algorithms can be considered for LDA. Popular methods are mean-field methods and collapsed Gibbs sampling (Porteous et al., 2008). However, both of these methods require a rederivation of the inference method when applied to new topic models, which can be time-consuming. This drawback has been the basis for black-box inference methods, which require only very limited and easy to compute information from the model and can be applied automatically to new models (Srivastava and Sutton, 2017). Autoencoding variational Bayes (AEVB) is a natural choice for topic models as it trains an inference network (Dayan et al., 1995); a neural network that directly maps the BOW representation of a document onto a continuous latent representation. A decoder network then reconstructs the BOW by generating its words from the latent document representation (Kingma and Welling, 2014). ProdLDA and NeuralLDA are the first topic modeling algorithms that use AEVB inference methods. In ProdLDA, the distribution over individual words is a product of experts (it models a probability distribution by combining the output from several simpler distributions) rather than the mixture model used in NeuralLDA.

##### 2.3.2.3. ETM

Another problem with LDA is dealing with large vocabularies. To fit good topic models, practitioners must severely prune their vocabularies, typically done by removing the most and least frequent words. To this end, the embedded topic model (ETM) is proposed (Dieng et al., 2020). ETM is a generative model of documents that combines traditional topic models with word embeddings. The ETM models each word with a categorical distribution whose natural parameter is the inner product between the word's embedding and an embedding of its assigned topic.

##### 2.3.2.4. CTM

The topic models described above should all be trained on unilingual datasets. However, many data sets (e.g., reviews, forums, news, etc.) exist in multiple languages in parallel. They cover similar content, but the linguistic differences make it impossible to use traditional, BOW-based topic models. Models have to be either unilingual or suffer from a vast but highly sparse vocabulary. Both issues can be addressed with transfer learning. The cross-lingual contextualized topic model (CTM), a zero-shot cross-lingual topic model, learns topics in one language and predicts them for unseen documents in different languages. CTM extends ProdLDA and is trained with input document representations that account for word-order and contextual information, overcoming one of the main limitations of the BOW models (Bianchi et al., 2020).

##### 2.3.2.5. HDP

A different topic modeling algorithm based on the Dirichlet distribution is the Hierarchical Dirichlet Process (HDP), which is a Bayesian non-parametric model that can be used to model mixed-membership data with a potentially infinite number of components. In contrast to all the algorithms discussed in this section, HDP is the only algorithm that determines the number of topics itself (rather than being set by the user). Given a document collection, posterior inference is used to determine the number of topics needed and to characterize their distributions (Wang et al., 2011).

## 3. Study Design

In this section, we provide the details of our comparative study. We describe first the dataset that we have used, followed by the training of the topic models. Then, we explain the classifier we used. Finally, we provide details of our comparison and evaluation methodology,

### 3.1. Data

The data for this research consists of clinical notes, written in Dutch, by nurses and physicians in the University Medical Center (UMC) Utrecht's psychiatry ward between 2012-08-01 and 2020-03-01 as used in previous studies (Mosteiro et al., 2020, 2021; Rijcken et al., 2021). The 834,834 notes available are de-identified for patient privacy using DEDUCE (Menger et al., 2018b). Since the goal of the topic models is to increase the understanding of the decisions made by the subsequent text classification algorithm, we maintain the same structure as in previous studies. Each patient can be admitted to the psychiatry ward multiple times. In addition, an admitted patient can spend time in various sub-departments of psychiatry. The time a patient spends in each sub-department is called an admission period. In the data set, each admission period is a data point. For each admission period, all notes collected between 28 days before and 1 day after the start of the admission period are concatenated and considered as a single period note. We preprocess the text by converting it to lowercase and removing all accents, stop words and single characters. This results in 4,280 admission periods with an average length of 1,481 words. Admission periods having fewer than 101 words are discarded, similar to previous work (Menger et al., 2018a, 2019; Van Le et al., 2018). The dataset is highly imbalanced: amongst the 4,280 admission periods, 425 and 3,855 are associated with violent- and non-violent patients, respectively.

### 3.2. Training Topic Models

For the comparison of topic models, we have used the OCTIS Python package (Terragni et al., 2021) and FuzzyTM^2^. In total, we train and compare 17 different algorithms: LDA, NeuralLDA, ProdLDA, NMF, CTM, ETM, LSI, HDP, and three variations for each FLSA, FLSA-W, and FLSA-V. The three variations for the FLSA-based algorithms differ in the fuzzy clustering algorithms used. We apply fuzzy c-means clustering (Bezdek, 2013), FST-PSO clustering (Nobile et al., 2018; Fuchs et al., 2019), and Gustafson-Kessel clustering (Gustafson and Kessel, 1979). Since the number of topics can influence a topic model's coherence significantly (Rijcken et al., 2021), we train all the topics models with five to 100 topics in steps of five. Since HDP automatically selects the number of topics, we did not include this in the grid search for number of topics. To account for randomness in topic model initialization, we run each combination of topic models with the number of topics ten times. Consequently, we trained a total of 3,210 topic models (16 algorithms with 20 models plus the HDP model^3^, make a total of 3,210 topic models).

### 3.3. Classification Model

We use two different approaches to create a topic embedding for each document. For the first approach, we include all the words of a topic's distribution and use the vectors from *P*(*T*|*D*) for the classification of each document. We found 20 words to be most interpretable in previous research (Rijcken et al., 2021). For the second approach, we use a topic embedding approach based on the top *n* words per topic, since topics are typically represented by the top-*n* words. Hence, we also use *n* = 20 in this paper. For each topic, the probabilities associated with the words that are present in both the document and the topic's top-*20* words are aggregated.

**Figure 1:**
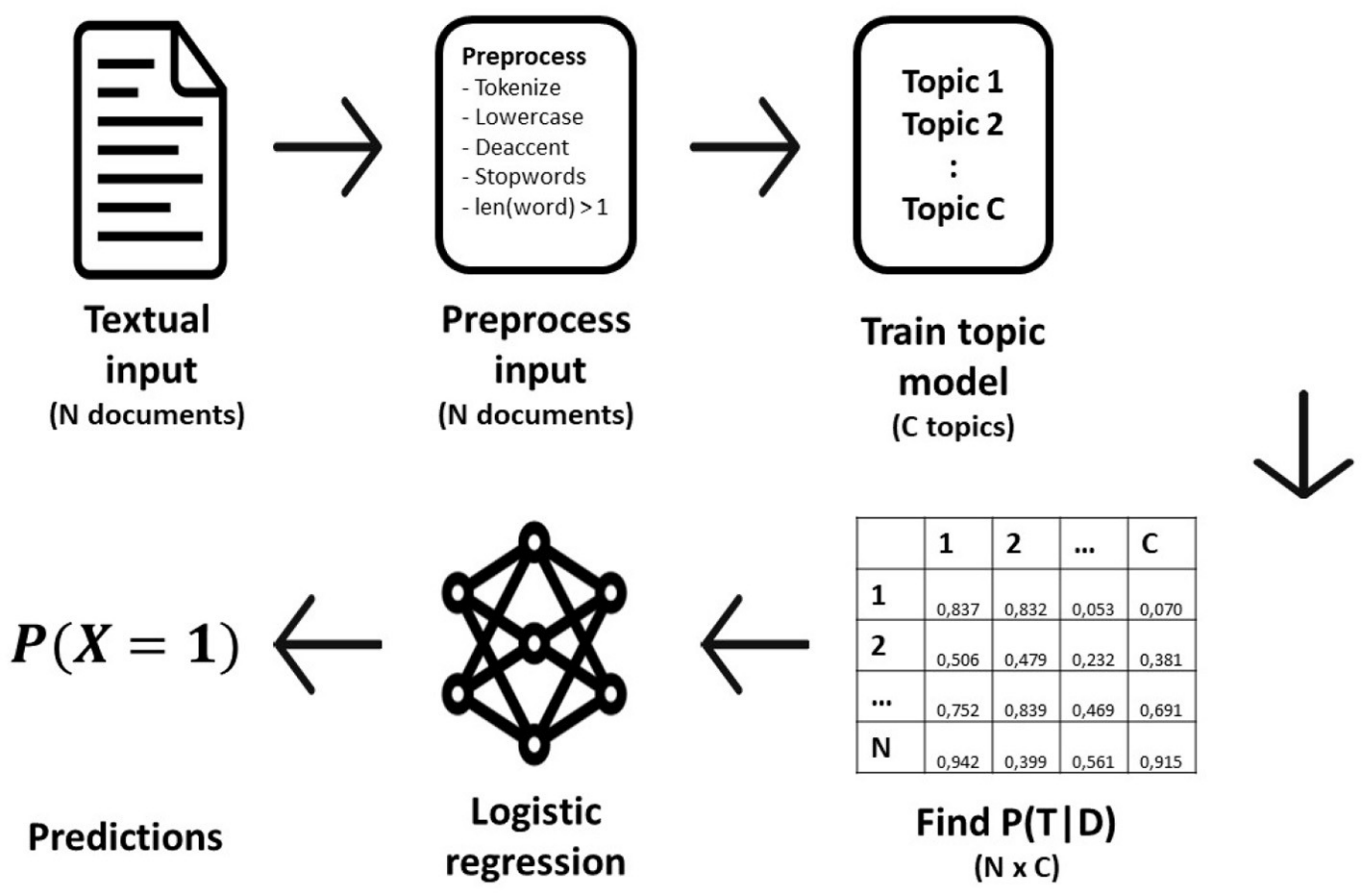
Visual representation of the modeling pipeline per algorithm.

There are many machine learning methods that can be used for classification. One of the most popular and simplest models for binary prediction is logistic regression. In this paper, we use logistic regression with 10-fold cross validation as the prediction model because of its simplicity and fast training time. A visual impression of the modeling pipeline is depicted in Figure 1.

### 3.4. Evaluation

The evaluation of the topic models depends on the evaluation goals, which are operationalized through various metrics. In this paper, we consider the quality and the prediction performance of the topic model obtained by using the topic model as the criteria along which we evaluate different algorithms. The ideal way for evaluating the quality of a topic model is human evaluation. Various methods have been suggested for this purpose (Chang et al., 2009). However, human evaluation is costly in terms of effort and is not feasible with a large number of models. Since we are training and comparing 3,210 models, we use quantitative measures for comparison. In particular, we use an interpretability score and classification performance as the aspects along which our comparison is made. In this section, we explain the definition of these metrics.

#### Interpretability Score

Interpretability is an abstract concept that could be considered along a number of aspects. From the perspective of modeling from EHR data, our interactions with the clinicians have shown that two aspects are very important. Firstly, the words within each topic (intra-topic assessment) must be semantically related. We use the coherence score (*c*_*v*_) to quantify this. Secondly, different topics should focus on different themes and be diverse; for this, we use the diversity score (inter-topic assessment). Then, we formulate the interpretability score as the product between coherence and diversity, similar to Dieng et al. (2020).

Amongst the quantitative measures for intra-topic assessment, such as perplexity or held-out likelihood, methods that are based on Normalized Pointwise Mutual Information (NPMI) correlate best with human interpretation (Lau et al., 2014). One measure that incorporates NPMI is the coherence score. This score indicates how well words support each other and the score can be divided into four dimensions: the segmentation of words, the calculation of probabilities, the confirmation measure and the aggregation of the topic scores. Röder et al. (2015) tested each possible combination on six open data sets and compared it to human evaluation. Based on this extensive setup, *c*_*v*_ was found to correlate highest with human evaluation, amongst all the coherence scores. With *c*_*v*_, the Normalized Pointwise Mutual Information (2) is calculated for the combination of all the top-*n* words in a topic. For the calculation of the NPMI, a sliding window of size 110 is used to calculate the probabilities. Then, the arithmetic mean is calculated to aggregate the scores for different topics.


(1) $$\begin{array}{rcl} PMI\left(w_{i},w_{j}\right) & = & log\frac{P\left(w_{i},w_{j}\right)+\epsilon }{P\left(w_{i}\right)\cdot P\left(w_{j}\right)} \end{array}$$

(2) $$\begin{array}{rcl} NPMI{\left(w_{i},w_{j}\right)}^{\gamma } & = & {\left(\frac{log\frac{P\left(w_{i},w_{j}\right)+\epsilon }{P\left(w_{i}\right)\cdot P\left(w_{j}\right)}}{-log\left(P\left(w_{i},w_{j}\right)+\epsilon \right)}\right)}^{\gamma } \end{array}$$

The coherence score ranges between zero and one, where one means perfect coherence and zero means no coherence whatsoever. Since the coherence score focuses on the support of words within topics, it only focuses on intra-topic quality and ignores inter-topic quality.

A measure for inter-topic quality is topic diversity (Dieng et al., 2020), which measures the unique words in a topic model as a proportion to the total number of words (3). Mathematically, we calculate the topic diversity as follows. Let W^*^ be the set of top-*n* words that have been identified for *C* topics. Then, the diversity score *D* is defined as


(3) $$\begin{array}{r} D=\frac{\left|W^{*}\right|}{nC}. \end{array}$$

If the topic diversity equals one, different topics do not share any common words, whereas a value of $\frac{1}{C}$ indicates that all topics contain the same *n* words.

#### Predictive Performance

To assess the predictive performance of the topic models, we use both the area under the ROC curve (AUC) (Fawcett, 2006) and the area under the Kappa curve (AUK) (Kaymak et al., 2012). The AUC is one of the most commonly used scalars for ranking model performance and was used in previous work as well (Mosteiro et al., 2020), (Menger et al., 2018a; Mosteiro et al., 2021). The AUC is independent of class priors, but it ignores misclassification costs. For this problem of violence risk assessment, misclassification costs may be asymmetric since having false positives is less problematic than having false negatives. The AUK is based on Cohen's Kappa (Cohen, 1960) and corrects a model's accuracy for chance agreement. The main difference between AUC and AUK is that AUK is more sensitive to class imbalance than AUC.

## 4. Results

**Figure 2:**
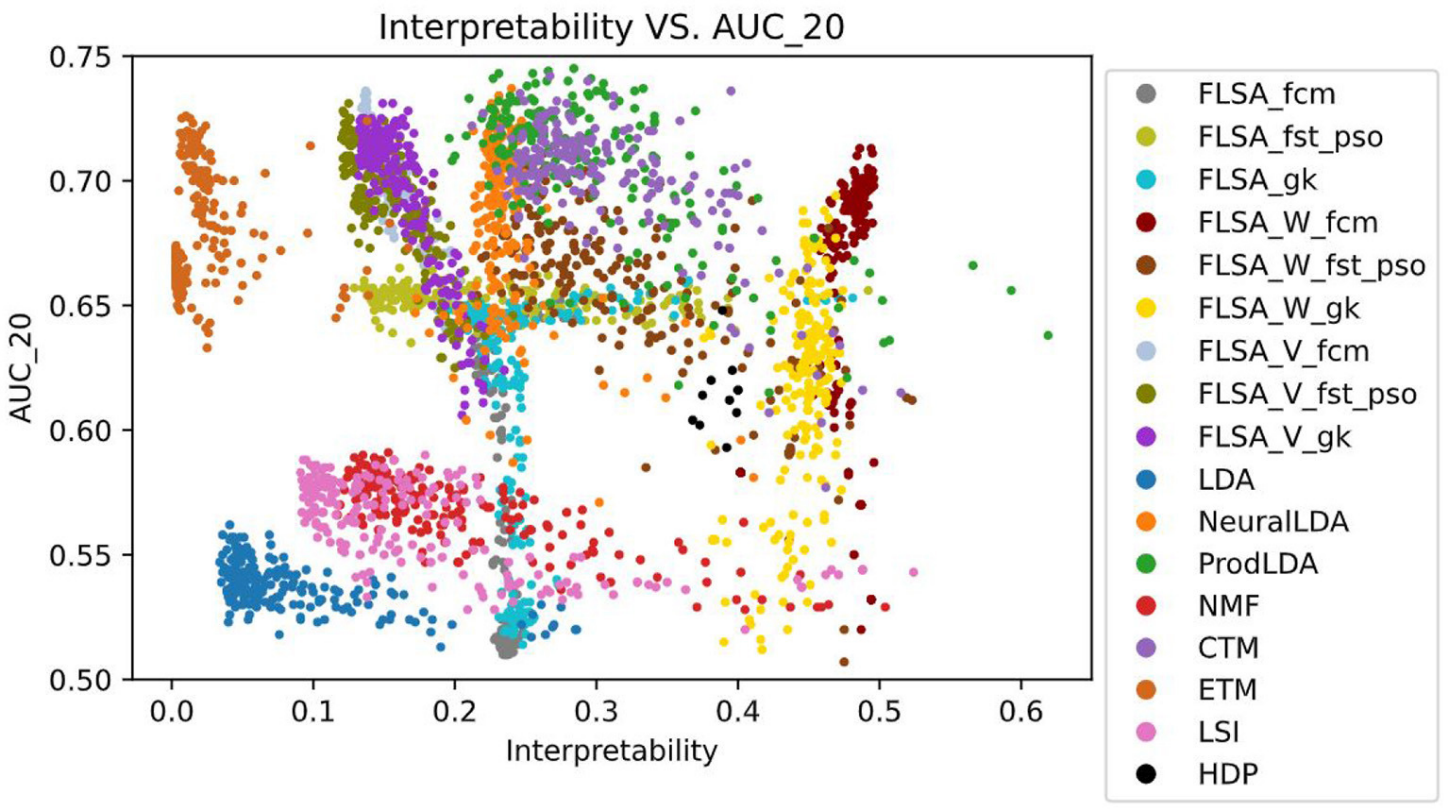
Model interpretability vs. predictive performance per trained model, as measured by the AUC_20 (only the top 20 words per topic are considered.).

**Figure 3:**
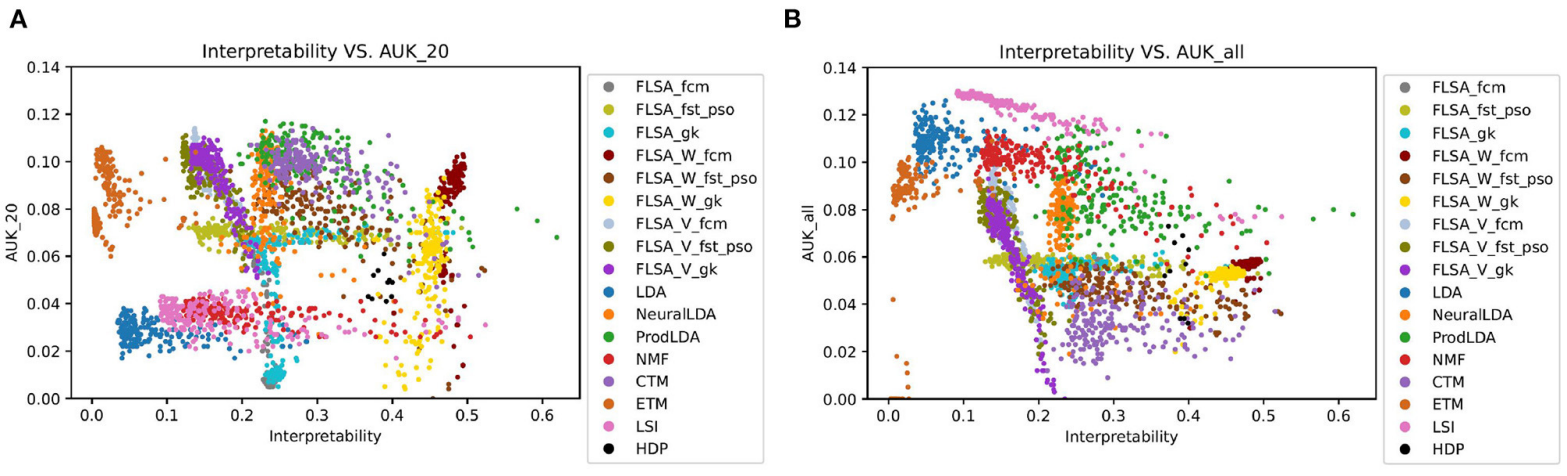
Two graphs showing how a model's interpretability relates to its predictive performance, as measured by the AUK per trained model. **(A)** Shows predictions based on a topics first 20 words only, while **(B)** takes an entire topics' distribution into account.

Figure 2 shows the performance (AUC) against the interpretability index for the trained topic models. Figure 3 shows the same, but the predictive performance is measured by the AUK for both the top-20 words and the entire topic distribution. The subscript of each performance metric indicates the number of words considered for the prediction: *20* means the top 20 words only and *all* means the entire topic distribution is considered. The patterns in Figures 2, 3 look similar because the Kappa curve is a nonlinear transformation of the ROC curve, but there are also differences. For example, LSI results are clearly separated from LDA results according to AUC and interpretability, but the separation is much smaller when considering AUK. This is because AUK indicates that the performance of LSI and LDA models is more similar than what is indicated by AUC.

Therefore, we will focus on the AUC only for the rest of this analysis. From the graphs, it can be seen that there is no correlation between a model's interpretability and predictive power. Also, no model outperforms other models for both indicators, for all parameter settings. When basing the predictions on the top 20 words per topic only, FLSA-W (with fcm clustering) and ProdlDA seem to perform best. ProdLDA has many instances with the highest predictive performance (and average interpretability) and a few instances with the highest interpretability (and suboptimal predictive performance). In contrast, almost all instances of FLSA-W have high predictive performance and high interpretability, but no instance has a maximum for either of the variables. It seems that FLSA-W operates at a different trade-off point between performance and interpretability than ProdLDA.

**Figure 4:**
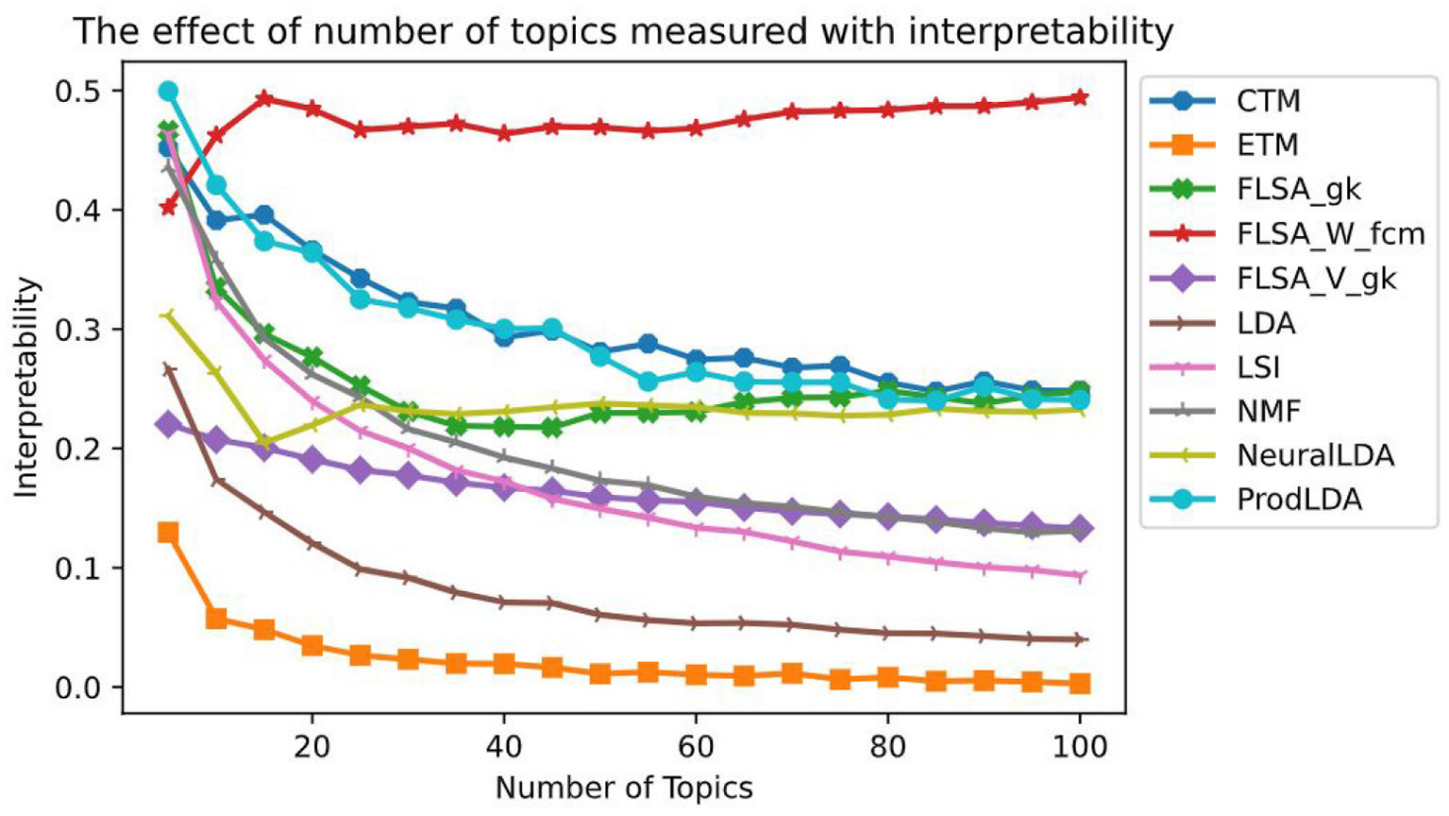
The effect of number of topics on the Interpretability—each data point is a mean score based on 10 runs.

**Figure 5:**
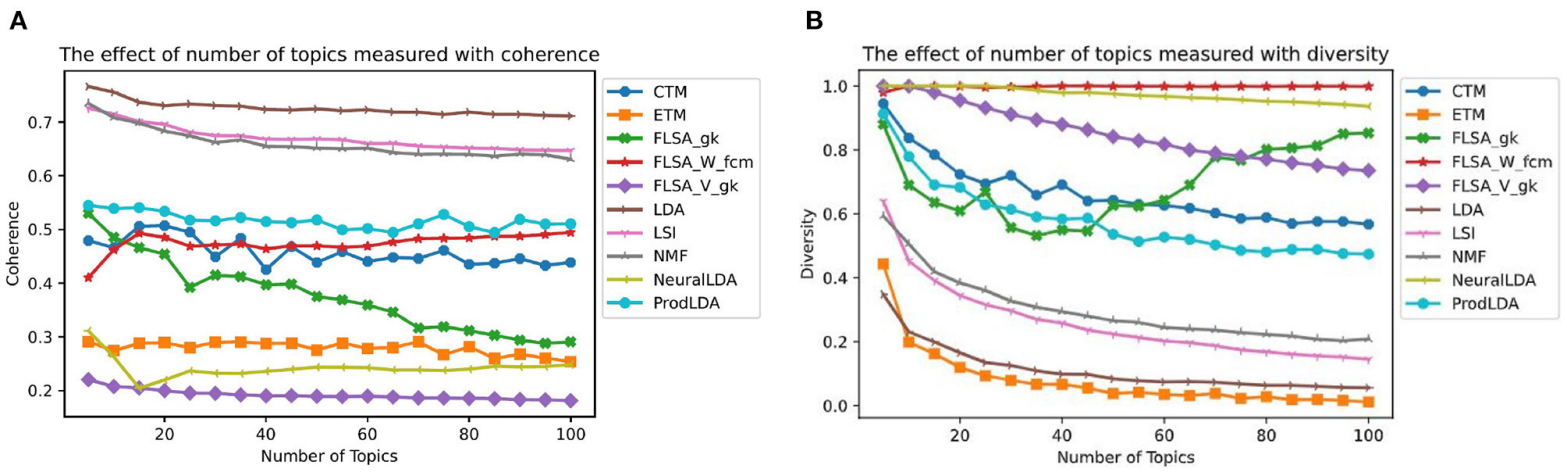
The effect of number of topics on the coherence, based on the top 20 words **(A)** and diversity **(B)**—each data point is a mean score based on 10 runs.

Figure 4 shows for each model the effect of the number of topics interpretability. Each data point in this graph is the average of ten models trained with that setting. We only show each FLSA model with its best clustering method for clarity. For each FLSA model, we selected the clustering method that scored highest on interpretability on the most number of topics amongst the other clustering methods. To allow for comparison, we keep these settings for the following graphs. Except for the lowest number of topics, FLSA-W (with FCM clustering) scores best on interpretability on all numbers of topics. Since the interpretability consists of both the coherence- and the diversity score, both give relevant insights. Figure 5 shows the graphs for these variables. It can be seen that LDA scores the highest on topic coherence and the second-lowest on diversity. This means that the words in the topics support each other but that many topics share most of their words. FLSA-W has almost a perfect diversity score for all topics, and its coherence score is average. ProdLDA's coherence score is slightly higher than LDA, but its diversity is much lower and decreases as the number of topics increase.

**Figure 6:**
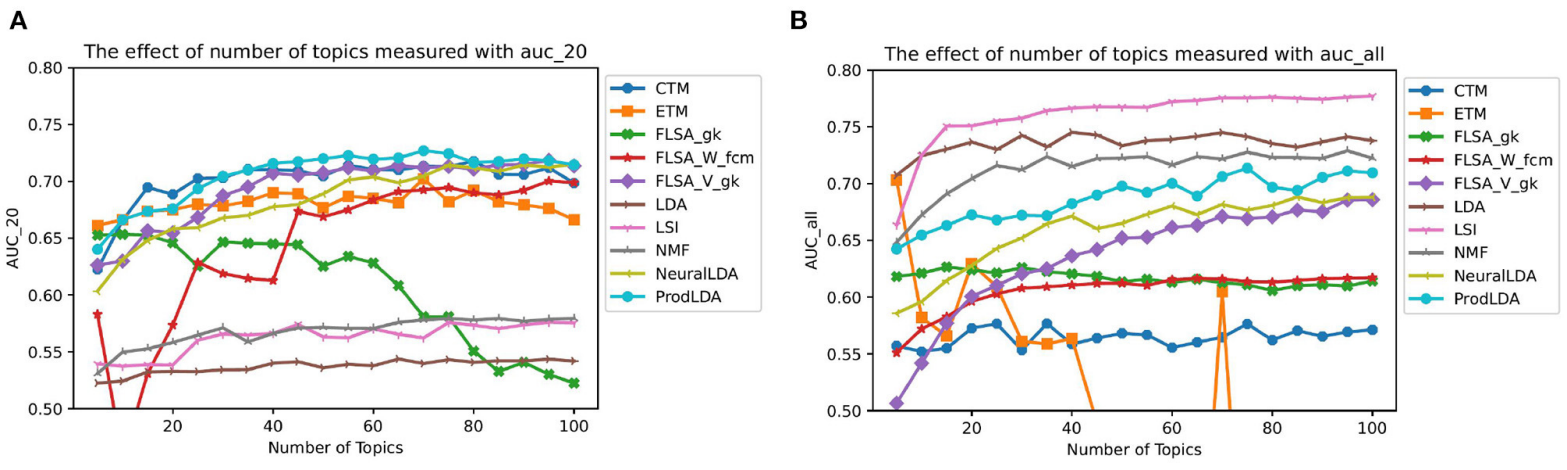
The effect of number of topics on AUC. **(A)** Shows predictions based on 20 words only and **(B)** shows predictions based on the entire topic distribution—each data point is a mean score based on 10 runs.

Figure 6 shows the effect of different number of topics on the predictive performance. Note that for the FLSA-based models the clustering methods with the highest interpretability scores are shown only. Since the interpretability and predictive performances do not correlate, these models do not necessarily have the highest predictive performance. The graph shows that some models' predictive performance is better when the top-20 words are considered only (CTM, FLSA-W, FLSA-V, ProdLDA, and NeuralLDA). In contrast, other models perform better when the entire topic distribution is considered (LDA, LSI, NMF). ProdLDA has the highest predictive performance based on the top 20 words only, whereas LSI has the highest predictive power based on the entire topic distribution.

## 5. Discussion

We study the behavior of different topic modeling algorithms based on their interpretability and predictive performance. LDA was used as a topic embedding in earlier text classification approaches with topic embeddings. However, Figure 2 shows that LDA has the lowest interpretability and predictive performance amongst all topic models. Although LDA has high coherence scores, many topics contain the same words, and therefore the interpretability is low. Our results show that selecting a topic model for text classification is not straightforward as no model outperforms the other models both in interpretability and predictive performance. If the interpretability needs to be maximized, FLSA-W is the new preferred model based on the interpretability index, whereas ProdLDA or LSI are preferred for maximal predictive performance.

Whether ProdLDA or LSI are preferred depends on the number of words per topic to base the predictions on. We argue that for the sake of interpretability, predictions based on a topic's top-*n* (20 in our case) words are preferred over the ones based on the entire distribution on two counts. Firstly, we use topic embeddings for interpretable text classification. It is more intuitive to interpret a set of *n* words, than it is to interpret complete distributions where all distributions contain the same words, but the probabilities per word vary. Secondly, no meaningful coherence score can be calculated on a full distribution as the coherence considers the words of a topic and not the probability. If the full topic distribution is taken, the coherence score would be the same for all topic models. Note that, the best predictive performance of the LSI model, based on all words, performs almost on par (with the AUC slightly below 0.8) with the best predictive performance in earlier work (Mosteiro et al., 2021), and hence, we recommend considering topic embeddings for future text classification approaches is reasonable.

**Figure 7:**
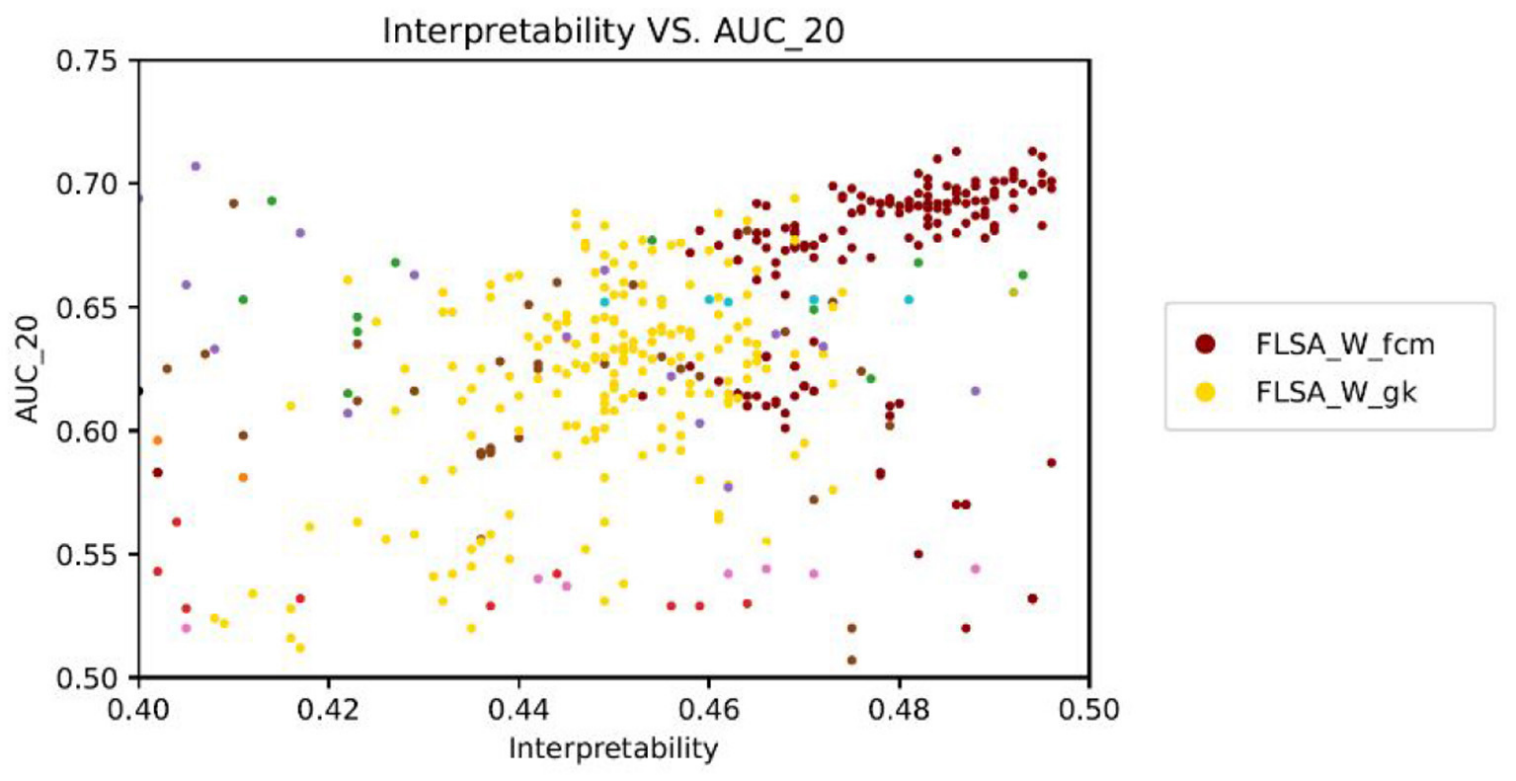
Interpretability vs. AUC_20 zoomed in FLSA_W_FCM and FLSA_W_GK shows a positive correlation between interpretability and predictive performance - each data point represents a trained model.

Surprisingly, Figure 2 which is based on all models, shows no correlation between model interpretability and predictive performance. Figure 7 shows the same data as Figure 2 but zoomed in on FLSA_W_FCM and FLSA_W_GK. We observe a positive correlation between the two variables for these two models. In contrast, ETM, NMF, LSI, LDA, FLSA_W_fst_pso, FLSA_V_fst_pso, FLSA_V_fcm show a slightly negative correlation between interpretability and predictive performance. The lack of correlation between these two variables raises the question of what information the classifier uses for its decisions. If words in topics do not support each other, then a topic is considered noisy. Yet, the predictive performance of models is reasonably high, implying there might be some tension between topic coherence and the prediction ability of the models.

A limitation of our work is that we work with a private, specific and imbalanced dataset with relatively long texts. Therefore, it is unknown whether our results can be extended to other datasets. Another limitation is that we formulate interpretability as the product between a topic's coherence and diversity, based on recent work (Dieng et al., 2020). However, coherence does not always correlate with human interpretation (Rijcken et al., 2021). Furthermore, human evaluations could shine more lights on the topic interpretability, but this is infeasible in our current setup due to the high number of topic models that we have trained (3,210). Lastly, interpretability cannot be reduced to a single number as it is a complex concept, but using a single metric can serve as a proxy for topic comparison at large scale.

## 6. Conclusion

There are many applications of text classification based on electronic health records in the clinical domain. For these tasks, classification interpretability is imperative. Using topic modeling algorithms as topic embeddings for text classification might make a model more explainable. Therefore, this work studies both the topic's interpretability and predictive performance for interpretable text classification. Comparing all models, we have not found a model that outperforms the other models both on interpretability and on predictive performance. Based on our findings, the FLSA-W (fcm) seems to be the best model for interpretability, whereas ProdLDA seems the best choice for predictive performance. However, this finding is based on one dataset only, and future work should assess the generalizability to other datasets. We found no correlation between a model's interpretability and predictive performance. More specifically, we observed that some topic models' predictive performance correlate positively with interpretability, whereas others show an inverse correlation. Also, we found that some models' predictions are better when the entire topic distribution is used, whereas others score better with the top 20 words only. This work demonstrates that selecting a topic modeling algorithm for text classification is not straightforward and requires careful consideration. Future work will investigate based on which information classifiers make their decisions. This insight could explain why different models' predictive performance correlates differently with interpretability and why for some models, predictions are better when based on the entire distribution and others are better when based on the top-*n* words only. Since we assess a topic's interpretability quantitatively, future work should also focus on qualitatively assessing a topic's interpretability. If topics are found to be interpretable based on the qualitive assessment, the decisions made by the text classification algorithm are more interpretable. With interpretable algorithms, we are one step closer to implementing automated methods for violence risk assessment and other text classification tasks in the mental health setting.

## Data Availability Statement

The datasets presented in this article are not readily available because this is a dataset with pseudonymized clinical notes from the EHR. Requests to access the datasets should be directed to e.f.g.rijcken@tue.nl.

## Author Contributions

ER and UK planned the experiment. The experiment was carried out by ER. PM supported with the coding. The manuscript was written by ER, with support from UK, FS, MS, and KZ. The dataset was provided by FS. All authors contributed to the article and approved the submitted version.

## Conflict of Interest

The authors declare that the research was conducted in the absence of any commercial or financial relationships that could be construed as a potential conflict of interest.

## Publisher's Note

All claims expressed in this article are solely those of the authors and do not necessarily represent those of their affiliated organizations, or those of the publisher, the editors and the reviewers. Any product that may be evaluated in this article, or claim that may be made by its manufacturer, is not guaranteed or endorsed by the publisher.

## Footnotes

^1^Also referred to as Latent Semantic Analysis (LSA).

^2^ https://pypi.org/project/FuzzyTM/

^3^Each trained 10 times.
